# Predictors of functional dependency in Parkinson's disease

**DOI:** 10.1002/mds.26751

**Published:** 2016-10-06

**Authors:** Angus D. Macleod, Carl E. Counsell

**Affiliations:** ^1^ Institute of Applied Health SciencesUniversity of AberdeenForesterhillAberdeenUK

**Keywords:** Parkinson's disease, predictors, dependency, prognosis

## Abstract

**Background:**

Functional dependency, the need for help in basic activities of daily living, is an important patient‐oriented outcome. We aimed to describe the development of dependency in Parkinson's disease (PD) and identify independent prognostic factors for this outcome.

**Methods:**

We analyzed data from the Parkinsonism Incidence in North‐East Scotland (PINE) study, a prospective, community‐based incident cohort of PD with ongoing follow‐up. We described the development of dependency defined by a Schwab & England score of < 80% and a Barthel Index of <19. We identified the baseline predictors of dependency using multivariable Cox regression.

**Results:**

In 198 patients with PD, the rate of development of dependency was 14 per 100 person years. Older age at diagnosis (hazard ratio for 10‐year increase 2.23 [95% confidence interval 1.66‐2.98]), greater smoking history (hazard ratio for 10‐pack‐year increase, 1.15 [1.04‐1.26]), more severe axial impairment (hazard ratio for 5‐point increase in sum of axial items from UPDRS scale, 1.78 [1.30‐2.44]), and lower MMSE score (hazard ratio 0.88 [0.79‐0.98]) were independently associated with a higher risk of dependency as defined by Schwab & England. Only older age (hazard ratio for 10‐year increase 1.35 [1.04‐1.76]) and severity of axial impairment (hazard ratio for 5‐point increase 1.85 [1.31‐2.62]) were associated with a higher risk of dependency as defined by the Barthel Index. Sex, deprivation, comorbidity, overall UPDRS motor score, and disease stage were not independently associated with dependency.

**Conclusion:**

This is the first community‐based study of dependency in PD. There was a high rate of dependency development. Older age, more smoking, more axial impairment, and poorer cognition were independent predictors. © 2016 The Authors. Movement Disorders published by Wiley Periodicals, Inc. on behalf of International Parkinson and Movement Disorder Society.

Functional dependency is an important patient‐oriented outcome that has been infrequently studied in Parkinson's disease (PD). In this article, we focus on dependency in basic daily tasks, defined as being dependent on others for help with basic activities of daily living (ADLs), such as walking, washing, dressing, toileting, and feeding. We do not consider other levels of dependency, such as dependency in instrumental ADLs (such as the ability to go shopping, manage money, or drive).

In a previous systematic review, we highlighted the paucity of data on the progression to, and the predictors of, functional dependency.^1^ Only 2 previous studies have reported the predictors of dependency:^2^, ^3^ one was carried out before and during the early part of the levodopa era, and both were from specialist clinics and therefore unlikely to be representative of the general population with PD. Neither cohort recruited and followed patients from the beginning of their disease course, which is the optimal way to study prognosis.^4^

We therefore sought (i) to describe the development of dependency and (ii) to identify the independent predictors of the development of dependency in an incident population‐based cohort of PD.

## Methods

### PINE Study

We analyzed data from the Parkinsonism Incidence in North‐East Scotland (PINE) study, a prospective, community‐based incident cohort of parkinsonism with ongoing long‐term follow‐up in Aberdeen, UK. There were 2 incidence periods: an 18‐month pilot phase in an area with a population of 148,600, beginning November 2002 and a 36‐month main study phase in an area with a population of 317,357 people, beginning April 2006.^5^, ^6^, ^7^ We endeavored to identify all patients with a previously undiagnosed degenerative or vascular parkinsonian syndrome using multiple, overlapping strategies for case ascertainment, including writing to general practitioners and relevant hospital specialists asking them to refer suspected cases; hand‐searching neurology and geriatric referral letters; and searching general practice databases and hospital discharge data electronically. All of the patients who were referred or identified through the searches and who did not have a previous diagnosis of a parkinsonian disorder were invited to be seen by a neurologist with a special interest in movement disorders (or a supervised trainee) for assessment. We defined incident parkinsonism broadly as either 2 or more cardinal features (rest tremor, bradykinesia, rigidity, or unexplained postural instability). The only exclusion was drug‐induced parkinsonism (defined by normal dopamine transporter imaging or by complete resolution of parkinsonism after withdrawal of the relevant drug). All incident patients were invited to consent to long‐term annual follow‐up. The study was approved by the Multi‐Centre Research Ethics Committee for Scotland and conducted with the informed consent of the patients involved.

This analysis was restricted to patients with idiopathic PD. At baseline and at each annual review, diagnoses were reviewed on the basis of clinical history, examination, and imaging findings. PD was defined by the UK PD Brain Bank criteria^8^ insofar as follow‐up duration permitted the supportive criteria to be applied. The latest diagnoses (after follow‐up between 5 and 11 years), including postmortem data (available for 10% of the whole cohort), were used for diagnostic classification for this analysis.

### Outcome Definition

Functional dependency was defined by both the Schwab & England (S&E) scale^9^ and the Barthel index^10^ at annual follow‐up visits (some patients had interim visits, when clinically required, at which S&E data were also collected). For the survival analyses reported in this article, we used sustained dependency as the outcome so only patients who remained dependent for the rest of their follow‐up were defined as having dependency, and patients who were dependent at 1 visit but subsequently were independent were not classified as being dependent at that time point.

The S&E scale is an 11‐point scale of ability to perform activities of daily living, ranging from 100% (completely independent) to 0% (vegetative) in 10% increments. Dependency was defined as an S&E score less than 80% (80% = completely independent in most chores; 70% = not completely independent). Although the word *chores* is open to interpretation, we consistently interpreted this as basic ADLs at each time the scale was used. The S&E scale has been partly validated for use in PD as a measure of ADLs with evidence of satisfactory construct validity,^11^ adequate longitudinal validity,^12^ good test‐retest reliability,^13^ and adequate interrater reliability.^14^ However, its validity as a dichotomous measure of dependency or independency has not been established. Nevertheless, it does have face validity for this purpose. The S&E scale was assessed by clinicians and was available from all patients.

The Barthel Index is a 20‐point scale that rates the level of dependency for specific basic ADLs. Its validity and reliability have been demonstrated in many diseases,^10^ although only 1 small study has reported its validity in PD.^15^ Different levels have been used as a cutoff for defining dependency with this scale, but we have followed Shah's description^16^ of 20 as no dependency and 19 as “slight dependency” and used a score of 18 or less as our cutoff for dependency. In this study, the Barthel index was self‐reported and was available only from a subset of patients who consented to a higher level of study involvement.

### Predictor Variables

To avoid overfitting the model, we selected potential predictors measured at baseline (at diagnosis), which we thought were likely to be associated with both PD and dependency, on the basis of previously reported associations,^1^ a previous analysis of motor predictors in the PINE study,^17^ and clinical knowledge. We decided that age should be included in the model irrespective of statistical significance because of strong evidence from previous studies that it is associated with multiple prognostic outcomes. The other candidate baseline variables were selected using a backward stepwise selection process: sex, DepCat (an area‐based deprivation score),^18^ whether the patient lived alone, pack years of smoking history, Charlson comorbidity score,^19^ severity of bradykinesia (sum of bradykinesia items from the UPDRS motor score), severity of axial features (sum of axial items from the UPDRS motor score), UPDRS part III overall score, MMSE score, and Hoehn & Yahr stage. For 98 patients who developed sustained dependency (measured by the S&E scale), this represents an events‐per‐variable ratio of about 10, the recommended minimum.^20^ Condensing comorbidity data into a single index was necessary for statistical efficiency,^21^ but it does result in the loss of information about individual comorbidities.

### Statistical Analyses

Using each scale in turn to define dependency, we plotted Kaplan–Meier curves of survival free from sustained dependency, calculated median duration of independence, and rates of development of sustained dependency. Patients who were dependent at the time of diagnosis were excluded. Patients who died or were lost to follow‐up prior to becoming dependent were censored at the time they were last seen. Patients remaining alive and independent were censored at the time of their last visit up until 19 January 2015. Independent predictors of dependency were identified using Cox regression. First, univariable analysis was performed with each candidate predictor in turn. Then those variables with association with dependency (*P* < .2) were included in a backward stepwise regression model. A probability cutoff of .1 was used for the removal of variables from the model (other than age). Functional form was tested by assessing whether a 2‐power fractional polynomial provided a better fit.^22^ The proportional hazards assumption was examined using tests based on Schoenfeld residuals.^23^ The influence of individual observations on the models was assessed by calculating the likelihood displacement. One patient was excluded from the model of dependency defined by the S&E scale because of high influence on the model.

All statistical analyses were performed with Stata version 12.1 (StataCorp LP, College Station, Texas).

## Results

**Figure 1:**
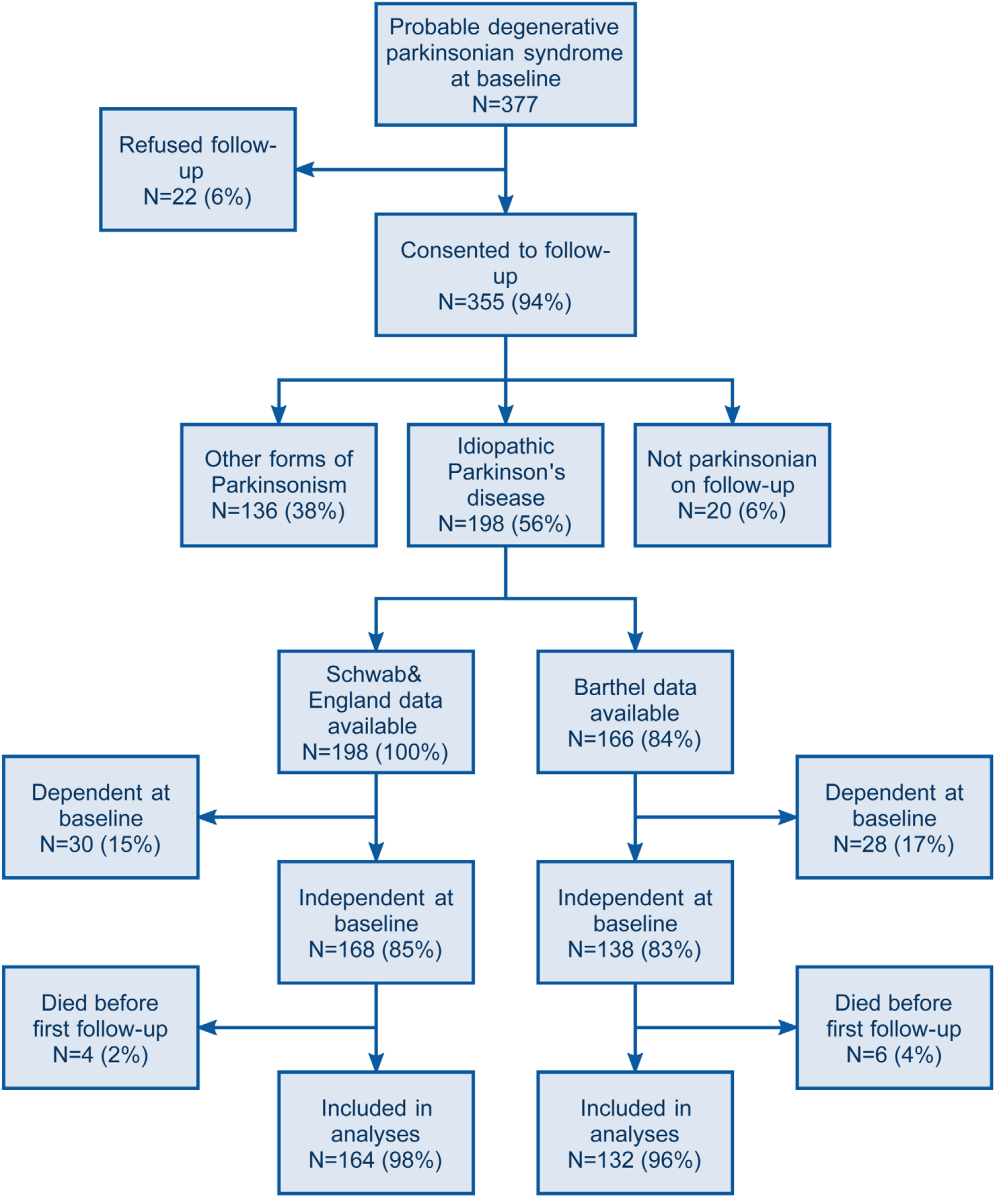
Flowchart of participation in the Parkinsonism Incidence in North‐East Scotland study. [Color figure can be viewed in the online issue, which is available at wileyonlinelibrary.com.]

A total of 198 patients with idiopathic PD consented to follow‐up (see Fig. 1 and Table 1). All patients had S&E data, but 32 (16%) did not consent to the higher level of study involvement, which included the Barthel index. Data were available with maximum possible follow‐up up between 5 and 12 years from diagnosis. Losses to follow‐up in the cohort were very low: No patient was lost to follow‐up regarding vital status, and only 2 patients were lost to follow‐up for S&E data.

**Table 1:** Baseline characteristics

Baseline variable	All patients (N = 198)	Patients with Barthel data available (n = 163)
Mean age in years at diagnosis (SD)	72.5 (10.4)	72.5 (10.0)
Number male (%)	119 (60)	97 (60)
Median symptom duration in months (IQR)	13 (9‐24)	17 (10‐24)
Mean H&Y stage (SD)	2.3 (0.8)	2.3 (0.8)
Mean UPDRS motor score (SD)	25.1 (11.6)	25.4 (11.6)
Mean MMSE (SD)	28.1 (2.3)	28.2 (2.1)
Median Charlson score (IQR)	1 (0‐2)	1 (0‐2)

Of the patients, 30 had sustained dependency from baseline as defined by S&E and 28 as defined by Barthel and were excluded from the analyses. A further 4 and 6 patients were excluded from the analyses defined by S&E and Barthel, respectively, because they were independent at baseline and died before their first follow‐up visit. Thus 162 patients were included in the analyses using the S&E data and 134 patients were included in the analyses using the Barthel data. Those who were dependent at baseline were older (eg, in the analyses using S&E data, mean age 76.3 vs 71.5 years) but of similar sex distribution (19% of men and 21% of women).

**Figure 2:**
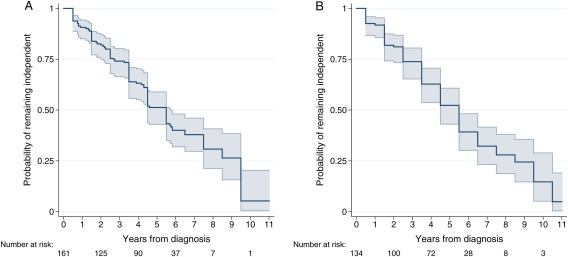
Kaplan–Meier probabilities of remaining independent with dependency defined in (**A**) by the Schwab & England scale and (**B**) by the Barthel Index. [Color figure can be viewed in the online issue, which is available at wileyonlinelibrary.com.]

Probabilities of remaining independent defined by the 2 scales were similar (Fig. 2). Kaplan–Meier probabilities of being dependent by 10 years of follow‐up were nearly 100%. The rate of development of sustained dependency (defined by S&E) in PD was 13.8 per 100 person years of follow‐up (95% confidence interval [CI] 11.3‐16.8) and the median duration of independent living after diagnosis of PD was 5.5 years (95% CI 4.5‐5.8). (These figures were very similar when calculated using the Barthel Index to define dependency.) Within individuals, from year to year there was more variability in Barthel scores than S&E scores.

Older age at diagnosis, greater smoking history, more severe axial impairment, and a lower MMSE score were independently associated with a higher risk of dependency development defined by the S&E scale; and in analyses of dependency defined by the Barthel Index, only older age at diagnosis and severity of axial impairment were associated with a higher risk of dependency (Table 2).

**Table 2:** Associations between baseline characteristics and dependency

	Dependency defined by Schwab & England < 80%				Dependency defined by Barthel score <19			
Baseline variable	Univariable association with dependency		Multivariable association with dependency^a^		Univariable association with dependency		Multivariable association with dependency^b^	
	Hazard ratio (95% CI)	*P* value	Hazard ratio (95% CI)	*P* value	Hazard ratio (95% CI)	*P* value	Hazard ratio (95% CI)	*P* value
Age at diagnosis (10‐year increase)	2.57 (1.95‐3.39)	<.001	2.23 (1.66‐2.98)	<.001	1.53 (1.19‐1.97)	.001	1.35 (1.04‐1.76)	.02
Sex (male vs female)	1.22 (0.82‐1.83	.32	0.99 (0.66‐1.50)	.97	1.15 (0.74‐1.78)	.53	1.07 (0.69‐1.67)	.76
DepCat deprivation score	0.99 (0.87‐1.12)	.87	0.96 (0.84‐1.10)	.57	0.99 (0.86‐1.13)	.84	0.97 (0.85‐1.12)	.70
Smoking history (10‐pack‐year increase)	1.18 (1.08‐1.28)	<.001	1.15 (1.04‐1.26)	.003	1.04 (0.95‐1.13)	.38	1.01 (0.92‐1.10)	.91
Charlson score (1‐point increase)	1.24 (1.08‐1.43)	.03	1.09 (0.93‐1.27)	.31	1.10 (0.91‐1.34)	.32	1.02 (0.84‐1.23)	.87
Bradykinesia score (5‐point increase)	1.54 (1.24‐1.90)	<.001	1.21 (0.88‐1.66)	.24	1.50 (1.18‐1.91)	.001	1.14 (0.80‐1.62)	.47
Axial score (5‐point increase)	1.28 (1.69‐3.06)	<.001	1.78 (1.30‐2.44)	<.001	2.09 (1.51‐2.90)	<.001	1.85 (1.31‐2.62)	<.001
UPDRS motor score (10‐unit increase)	1.55 (1.28‐1.87)	<.001	1.11 (0.77‐1.59)	.59	1.44 (1.17‐1.78)	.001	0.91 (0.56‐1.40)	.67
MMSE score (1‐point increase)	0.78 (0.70‐0.87)	<.001	0.88 (0.79‐0.98)	.02	0.85 (0.75‐0.96)	.009	0.95 (0.83‐1.08)	.41
Hoehn & Yahr stage	1.80 (1.33‐2.42)	<.001	0.95 (0.63‐1.43)	.82	1.66 (1.21‐2.29)	.002	1.06 (0.66‐1.68)	.82

CI, confidence interval.

Adjusted for variables in final model: age at diagnosis, smoking history, axial score, and MMSE score.

Adjusted for variables in final model: age at diagnosis and axial score.

## Discussion

In summary, in this incident cohort, almost all of the patients with PD were functionally dependent by 10 years of follow‐up. Median duration of independent living was 5.5 years. Because of the study design, this was a predominantly elderly cohort (mean age at diagnosis 72.5), with relatively few young‐onset patients included. Age, greater smoking history, more severe axial impairment, and worse cognition were independent early predictors of greater dependency. Fewer predictors of dependency were identified using the Barthel Index than S&E. This may be because of more year‐to‐year variability in Barthel scores, which may be because of the Barthel Index being self‐reported or because not all of the Barthel Index relates to ADLs (eg, incontinence), although there was also slightly lower power for the Barthel analyses.

Few previous studies have studied dependency in PD. One longitudinal study reported dependency using the S&E scale and found a lower risk of dependency than in our study (68% at after a mean 11 years of follow‐up).^24^ A study that defined dependency using clinical history‐taking also found a lower risk of dependency than in the PINE study (56% at 10 years),^3^ but another reported a higher risk of dependency (56% at 4 years).^25^ It is likely that selection biases and methodological differences explain the variation in the rates of dependency rather than true population differences in dependency risk. None of these other studies were inception studies (recruiting all patients from near their diagnoses) or community‐based studies. No previous study has reported longitudinal dependency data derived from the Barthel Index.

No population‐based or inception studies have previously reported risk factors for dependency. Only 2 studies, specialist‐clinic‐based noninception studies, have reported predictors for increased dependency (unlike this study, not measured specifically in early disease). One of these^2^ identified male sex, older age, akinesia rather than tremor, and no response to levodopa (which cannot be assessed at baseline) as independent risk factors. However, this study recruited patients before atypical parkinsonian syndromes were distinguished from PD, and the paper did not report hazard ratios for the prognostic factors. The other study^3^ reported that dementia, higher Hoehn & Yahr stage, worse ADL score, and several aspects of the UPDRS scale (rigidity, bradykinesia, postural instability, dyskinesia, and total score) were prognostic factors. However, these prognostic factors were only independent of age and disease duration and not independent of the other prognostic factors studied.

Age was reported as an independent predictor in 1 of these studies, but not in the other. However, age has consistently been reported as a prognostic factor for greater activity limitation in general.^26^, ^27^, ^28^ One study^3^ reported that dementia was associated with a greater risk of dependency, and other studies have identified MMSE as a predictor of greater disability in general.^26^, ^27^ No previous studies of prognostic factors for dependency or activity limitation investigated measures of axial impairment specifically, which was a stronger prognostic factor than the overall UPDRS score in this study.

We found that higher levels of smoking history were associated with more dependency, a finding that has not previously been reported. Although this finding was highly significant, it requires replication before we can be confident that it is not a chance finding. It may be a result of the effect of smoking‐related comorbidity contributing to dependency, although the baseline Charlson score was not associated with greater dependency, or perhaps may be due to smokers being more sedentary.^29^ This finding provides some evidence against a neuroprotective effect of smoking as has been suggested on the basis of lower incidence of PD in cigarette smokers than in nonsmokers^30^ and some experimental data from animal models of PD.^31^

It was surprising that comorbidity was not associated with greater dependency, and the only study that has investigated comorbidity burden as a prognostic factor in terms of disability in general found that it was independently predictive on 1 measure of activity limitation but not another.^26^ We cannot conclude from these data that comorbidity is not relevant to dependency because it may be that the burden of comorbidity at baseline is less important than the accrual of comorbidity after diagnosis in terms of the development of dependency.

The principal strengths of these analyses relate to the study design, in particular, the use of an inception cohort, an incident design, a very high consent rate to follow‐up, and very few losses to follow‐up; all of which should lead to low selection bias and high generalizability to the general population with PD. Other strengths related to the collection of dependency data—frequent collection (at least yearly), prospective collection, near‐complete data collection (for S&E data), and the use of 2 scales with clear face validity for identifying dependency—have led to a more complete description of the evolution of dependency in PD than previously reported. We have followed the principles advocated for the conduct of prognostic factor studies.^32^

There are also several limitations that deserve consideration. There was a lack of power to identify weak associations, and imperfect diagnostic accuracy is inevitable in any clinical study in PD.^33^ Neither scale has been validated as a measurement of dependency, although their validity as an activity limitation and disability measurement scale have been demonstrated. Yet, the similar probabilities dependent over time with both scales provide evidence for the construct validity of these scales to define dependency. There was internal consistency within the study team in the way the S&E scale was interpreted, that is, <80% if dependent on others for basic ADLs, but this may not be the universal interpretation. Although this may limit comparisons with raw S&E data from other studies, it should not limit the generalizability of the data on predictors of dependency. Because we tried to recruit all new patients in the population area, this is predominantly an elderly cohort, with few young‐onset patients (5% aged younger than 50 years). Our data are therefore mainly based on older‐onset PD, but we are unaware of any data to suggest that the effects of prognostic factors in PD vary by age.

This work has several benefits, both in clinical practice and for research. Defining the development of dependency and identifying its predictors are useful for providing better information to those affected by the disease. They can be combined into a prognostic model to provide individualized risk predictions.^34^ The development of dependency may also be a useful outcome measure in clinical trials because it is directly relevant to patients, is common early in the disease course in older cohorts, is relatively objective, and may be less confounded by dopamine replacement therapy than impairment measures. However, it may be less useful in young‐onset PD. Knowledge of prognostic factors can be used to enhance the design of clinical trials (eg, to stratify randomization) or as factors to adjust for in the analysis of trials or observational studies.^32^

In conclusion, we have described progression to dependency and predictors of dependency in a cohort with a low risk of bias. More research into dependency is needed (i) to identify which scale is best for measuring dependency, and how best to assess dependency in instrumental ADLs in PD (which may be more sensitive to early change, especially in younger patients); (ii) to combine the predictors together to develop and then validate a prognostic model that would provide additional value, both for individual risk prediction and for use in clinical trial design; and (iii) to evaluate the usefulness of dependency as an outcome in clinical trials.

## Author Roles

1. Research project: A. Conception, B. Organization, C. Execution; 2. Statistical Analysis: A. Design, B. Execution, C. Review and Critique; 3. Manuscript Preparation: A. Writing of the first draft, B. Review and Critique.

A.D.M: 1A, 1B, 1C, 2A, 2B, 3A

C.E.C.: 1A, 1B, 1C, 2C, 3B

## Full financial disclosures of all authors for the past 12 months

A.D.M. reports employment with the University of Aberdeen and grants from Chief Scientist Office of the Scottish Government (clinical academic fellowship), Parkinson's UK, NHS Grampian Endowments, and ISSF@Aberdeen (Wellcome Trust and University of Aberdeen). C.E.C. reports employment with the University of Aberdeen and grants from Parkinson's UK, Chief Scientist Office of the Scottish Government, NHS Grampian Endowments, and the Engineering and Physical Sciences Research Council.
